# The Secret to Successful Deep-Sea Invasion: Does Low Temperature Hold the Key?

**DOI:** 10.1371/journal.pone.0051219

**Published:** 2012-12-05

**Authors:** Kathryn E. Smith, Sven Thatje

**Affiliations:** University of Southampton, Ocean and Earth Science, National Oceanography Centre, Southampton, Southampton, United Kingdom; Ghent University, Belgium

## Abstract

There is a general consensus that today’s deep-sea biodiversity has largely resulted from recurrent invasions and speciations occurring through homogenous waters during periods of the Phanerozoic eon. Migrations likely continue today, primarily via isothermal water columns, such as those typical of Polar Regions, but the necessary ecological and physiological adaptations behind them are poorly understood. In an evolutionary context, understanding the adaptations, which allow for colonisation to high-pressure environments, may enable us to predict future events. In this investigation, we examine pressure tolerance during development, in the shallow-water neogastropod *Buccinum undatum* using thermally acclimated egg masses from temperate and sub-polar regions across the species range. Fossil records indicate neogastropods to have a deep-water origin, suggesting shallow-water species may be likely candidates for re-emergence into the deep sea. Our results show population level differences in physiological thresholds, which indicate low temperature acclimation to increase pressure tolerance. These findings imply this species is capable of deep-sea penetration through isothermal water columns prevailing at high latitudes. This study gives new insight into the fundamentals behind past and future colonisation events. Such knowledge is instrumental to understand better how changes in climate envelopes affect the distribution and radiation of species along latitudinal as well as bathymetric temperature gradients.

## Introduction

Throughout their evolutionary history marine invertebrates have colonised the oceans by extension of physiological boundaries. Phylogenetic links can be found between species in every ocean and throughout latitudinal and bathymetric ranges [1]. It is generally accepted that past colonisations to new latitudes have taken place during geological periods of cooling or warming (e.g. temperature declines during the Pliocene and Pleistocene eras) or through gradual adaptation to warmer or colder temperatures [2]–[4]. Patterns indicate such shifts to predominantly occur from the tropics, towards higher latitudes, with poleward declines in species diversity being observed throughout the water column [5]–[9]. Generally, invasions into, and emergences from, bathyal and abyssal depths are believed to primarily occur via isothermal water columns. Peak changes in diversity between shallow-water and deep-sea, appear in-line with certain geological periods when such isothermal waters were widespread (such as the Mesozoic and early Cenozoic eras), and more recently in high latitude areas through regions of deep-water formation [10]–[16]. Both fossil records and molecular phylogeny offer insight into such evolutionary paths. For example, Weddell Sea molluscs from the southern hemisphere can be tracked slowly north through fossil records from the Pliocene and Pleistocene periods [2] and close phylogenetic relationships have been proposed between shallow-water and deep-sea caridean shrimp [17], mytilid mussels [18] and anomuran decapods [19].

Many factors (biological, physical and chemical) affect the dispersal and colonisation of species [20], but for marine invertebrates, temperature and hydrostatic pressure create two substantial challenges. In shallow water temperature changes with latitude, and throughout the oceans both temperature and pressure change with depth. While both are known to affect biological systems throughout the whole animal, and are capable of causing major physiological disruptions, the impacts of these two variables have been found to be antagonistic, with pressure increases and temperature decreases often showing similar results [21]–[23]. A species combined tolerance to these two factors may therefore be vital in determining the upper and lower limits in its vertical distribution [24]. While peak changes in faunal assemblage are believed to have primarily occurred during past geological periods, new colonisations likely continue to take place today [3], [4], [13], [25], [26]. Current bathymetric migrations are being reported in areas with low water temperatures [13], [26], with recent studies indicating such events to be occurring in response to increasing surface water temperatures [27]–[29]. However, the growing amount of literature describing the combined effects of temperature and pressure on marine invertebrates typically indicates an increased sensitivity to pressure with decreasing temperature, with temperature being the dominant of the two factors. Work to date examining the combined effects of temperature and pressure on marine invertebrates typically focuses on echinoderms and crustaceans [12], [15], [24], [30]–[33]. Both adult and developmental stages have been investigated, but this includes only species with planktonic larvae. These studies generally exercise a small number of acute temperature and pressure treatments, with few studies examining the full physiological scope of an invertebrate with regard to both factors [32], [33]. Therefore, conclusions should not yet be drawn from the limited dataset available.

Understanding how the physiological scope of an organism is affected by thermal and hyperbaric changes may help us to realise the parameters that set its ecological boundaries. In turn this will aid us in predicting forthcoming bathymetric radiations, migrations and new evolutionary paths, which may occur within the oceans in response to the future effects of climate change [13]. In marine invertebrates, physiological tolerances often vary through ontogeny [e.g. 13,16]. A species combined tolerance throughout development is therefore key in dictating how successful future vertical migrations may be. In particular, it is important to understand where thresholds lie for species with non-planktonic development. Such species have limited dispersal capabilities, and migrations and radiations typically occur at a slower rate [34].

Neogastropods are a large group of molluscs, which are indicated by fossil records to have a cold, deep-water origin [35], [36]. Shallow-water species from this order may therefore be likely candidates for re-emergence into the deep sea. Species from certain families, including the Buccinidae, exist today throughout every ocean, from the intertidal to the abyssal zone [37]. Buccinidae regularly exhibit non-planktonic intracapsular development, the food-independent nature of which makes them ideal candidates for studying early ontogeny. Here, using *Buccinum undatum*, a widely distributed North Atlantic shallow-water buccinid, we examine the combined effects of temperature and pressure on early ontogeny. We use the novel approach of allowing thermal acclimation through development, and examining populations from different climates, across the species distribution range. Using respiration as an indicator of physical fitness, we investigate ecophysiological adaptations, which may indicate the potential for future deep-water colonisation.

## Materials and Methods

### Ethics Statement

All experiments were conducted in accordance with the legal requirements of the United Kingdom. The use of Molluscs is unregulated in the United Kingdom and subsequently does not require ethics approval by a specific committee. No specific permits were required for the described field studies.

### Study Species

The common whelk *B. undatum* is a shallow-water gastropod, which is found widespread across a latitudinal range running through the North Atlantic and Arctic Oceans. Across its range, it is annually exposed to temperatures ranging from below zero to above 22°C. Egg laying and intracapsular development occur across a narrower thermal range of 2 to 11°C [38], [39], although development is possible at temperatures up to 18°C [40]. Egg masses naturally take between 2.5 and 9 months to develop. At the southern end of its distribution this species is a winter spawner, with egg masses being laid as seawater temperatures cool and reach their lowest levels. In comparison, at the northern end of the distribution, or in colder waters, egg masses are laid in spring as water temperatures are warming [41]–[43]. *Buccinum undatum* is an important commercial species, providing locally valuable fisheries in several areas around the North Atlantic including the UK, the USA and Canada [44], [45]. Demand for this species is continuously increasing globally (Department of Marine Resources www.maine.gov/dmr/rm/whelks.html) and it has been suggested as an aquaculture candidate [46]. It has a close taxonomic relationship with several deep-sea species, for example *Buccinum abyssorum, Beringius turtoni, Belomitra quadruplex* [47] and *Buccinum thermophilium* [37].

### Egg Mass Collection

*Buccinum undatum* egg masses were collected from the Solent, UK (50°47′ N, 001°15′ W) between December 2009 and February 2010, and December 2010 and February 2011, and from Breiðafjörður, Iceland (65°00′ N, 023°30′ W) between April and May 2011. The methods of collection used are described below.

#### The Solent, UK

Egg masses were collected as described by Smith *et al*. [40]. In brief, collection took place using beam trawls deployed from on board *RV Callista* (5–10 m depth; water temperatures 4 to 10°C; www.bramblemet.co.uk/) or through farming in the seawater aquarium at the National Oceanography Centre, Southampton (water temperatures <8°C). For the latter, adult whelks were collected by Viviers from the Solent using whelk traps (∼15 m depth) (www.fishmarketportsmouth.co.uk). Egg masses were removed from aquarium walls 24 hours after laying had ceased.

#### Breiðafjörður, Iceland

Egg masses, which had been detached from substrate by heavy weather, were collected from the intertidal area by hand, from beaches around Breiðafjörður. During the collection period, seawater temperatures ranged from 3 to 4°C.

### Egg Mass Maintenance

Egg masses were maintained individually in 1.8 L incubation tanks containing aerated, 1 µm filtered seawater (three 100% water changes per week). Egg masses from the southern location (the Solent) were acclimated to 6, 10, 14 or 18°C (classified *n*°C (S)), and from the northern location (Breiðafjörður) to 3 or 6°C (classified *n*°C (N)). Temperatures were chosen because previous investigations on thermal tolerance during development have shown populations from the southern end of the distribution to be able to develop successfully under temperatures of 6 to 18°C [40]. Since these egg masses successfully developed at temperatures above, but not below natural developmental temperatures, it was assumed the same pattern would be observed from egg masses throughout the distribution. Therefore, a minimum temperature of 3°C was chosen for egg masses from the northern end of the distribution because this is the lowest developmental temperature observed in this area (Smith, personal observations). Three egg masses were maintained at each temperature. Acclimation was stepwise (1°C every 24 hours) from the initial water temperature at collection. Egg masses were maintained at each temperature throughout intracapsular development, and for a minimum of 21 days prior further pressure experimentation. Developmental timing was estimated using the results of Smith et al. [40] and ontogenetic stage, using the results of Smith & Thatje [39]. All experimental work was carried out at the National Oceanography Centre, Southampton, UK. Pressure experiments were carried out on two ontogenetic stages; veliger and hatching juvenile [39]. These stages were chosen as representative of the oldest and youngest ontogenetic stages, which could be manipulated without damage.

### Effects of Pressure on Respiration Rates

The effect of pressure on respiration rates was measured by transferring three individuals into a 2.8 ml plastic vial containing pre-incubated 1 µm filtered seawater. Veligers were sampled by gently flushing the content of an open capsule into a petri dish and transferring individuals by pipette. Individual veligers were randomly selected from three separate capsules for each vial. Juveniles were collected directly from the incubation tank walls. Vials were sealed under water to prevent air from being trapped inside. This allowed seawater to be pressurised without risking air being forced into the liquid, thus preventing the partial pressure of dissolved gases from being affected. A further two vials were filled containing animals, followed by four control vials, containing no animals. The seven vials were placed inside a pressure vessel (Stauff, UK), which was then filled with freshwater. Both the vessel and the freshwater were pre-incubated to experimental temperature. The vessel was pressurised to the selected experimental treatment (1, 100, 200, 300, 400 atm) using a Maximator model manual hydraulic pump [after 16,24]. Pressurisation was continuous and took a maximum of 30 seconds. Pressures ranging 1 to 400 atm (equivalent to 0 to 4000 m water depth) were chosen to represent shallow water through to average ocean depths. The pressure vessel was then incubated at the experimental temperature for 4 hours, before being rapidly depressurised [after 16,24]. Pressure remained continuous for the entire experimental period. Prior to experimentation, preliminary investigations were carried out to ascertain the duration of the experiment (4 hours), with regards to vial oxygen levels. Hypoxic conditions were considered to occur if oxygen levels fell below 60% air saturation and this figure therefore dictated experimental duration.

Upon depressurisation the oxygen concentration of the water (% air saturation) was recorded for each vial. Measurements were obtained using a temperature and atmospheric pressure adjusted oxygen meter and microoptode (Microx TX3, Presens, Germany). This equipment was calibrated daily using fully aerated seawater (100% O_2_ saturation), and seawater deoxygenated through oversaturation with sodium sulfite anhydrous (0% O_2_ saturation), both pre-incubated to temperature [24]. Each vial was gently agitated prior to the measurement being taken to ensure the oxygen level was constant throughout. The % air saturation was then continuously logged for a minimum of one minute or until a constant level of air saturation was observed. Readings from the control vials were averaged for further calculations. Animals from each vial were stored together in a pre-weighed (6 mm×4 mm) tin capsule and frozen at −80°C. Samples were freeze-dried over 24 hours and then whole-animal dry weight was determined (±1 µg).

The final respiration rate was calculated following the method used by Brown and Thatje [32], an adaptation of the protocol described by Thatje *et al*. [24]. Here, the difference in readings between control and experimental vials was determined and oxygen consumption assessed per unit of dry weight. This method accounts for any oxygen consumption occurring through microbial action. In this method, the concentration of oxygen in 100% saturated seawater was calculated according to Benson and Krause [48]. This protocol was repeated for every temperature/pressure combination, for 3 egg masses at each temperature, and for veliger and hatching juveniles from each egg mass.

### Statistics

Data were analysed using General Linear Model ANOVA (two factors, crossed; temperature and pressure). *Post hoc,* analysis was carried out using the Sidak simultaneous test. Prior to analysis all data were subject to square root transformation to attain homoscedasticity (Levenes test, p>0.05).

## Results

Oxygen consumptions recorded below, are stated as µmol O_2_ mg^−1^ h^−1^ (±1 S.D.).

### Effects of Temperature on Respiration Rates

**Figure 1:**
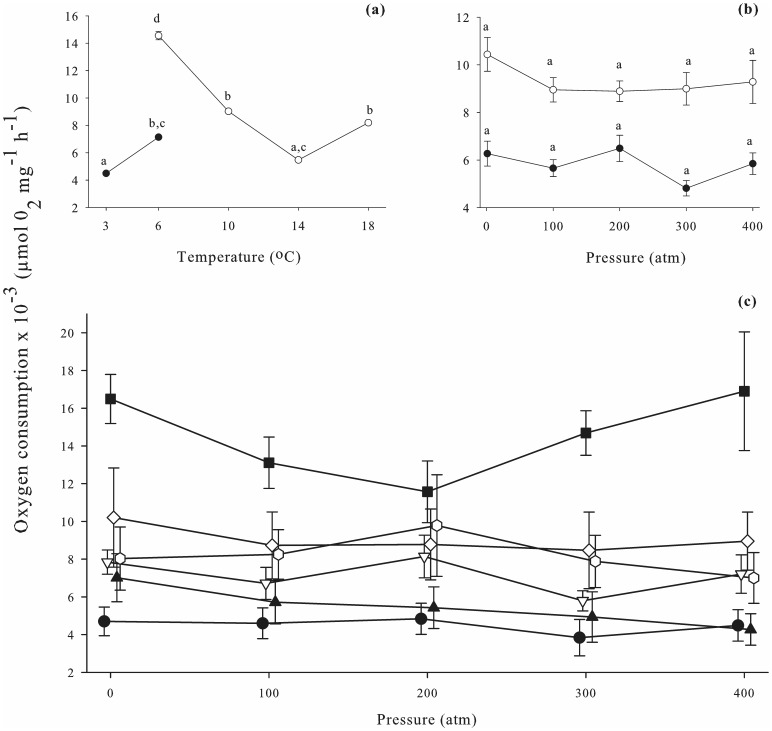
Oxygen consumption in *Buccinum undatum* veliger from the Solent (UK) and Breiðafjörður (Iceland). (a) Effects of temperature on oxygen consumption. Data for each temperature is averaged from 5 pressures (1, 100, 200, 300, 400 atm). Closed circles, Breiðafjörður data; open circles, Solent data. General Linear Model ANOVA indicated oxygen consumption to be significantly affected by temperature (*p≤*0.001); different letters indicate values that are significantly different. For each data point n = 45. (b) Effects of hydrostatic pressure on oxygen consumption. Data for each pressure is averaged across temperatures (6, 10, 14, 18°C for Solent samples; 3, 6°C for Breiðafjörður samples). Closed circles, Breiðafjörður data; open circles, Solent data. Oxygen consumption was not affected by pressure; different letters indicate values that are significantly different. For each data point n = 54. (c) Change in oxygen consumption with pressure, at 6 temperatures. Closed circles, 3°C Breiðafjörður; open circles, 6°C Breiðafjörður; closed triangles, 6°C Solent; open triangles, 10°C Solent; closed squares, 14°C Solent; open squares, 18°C Solent. Oxygen consumption was not affected by temperature – pressure interactions; see table 2 for *post hoc* analysis (Sidak simultaneous test). Error bars display standard error. For each point, n = 9.

**Figure 2:**
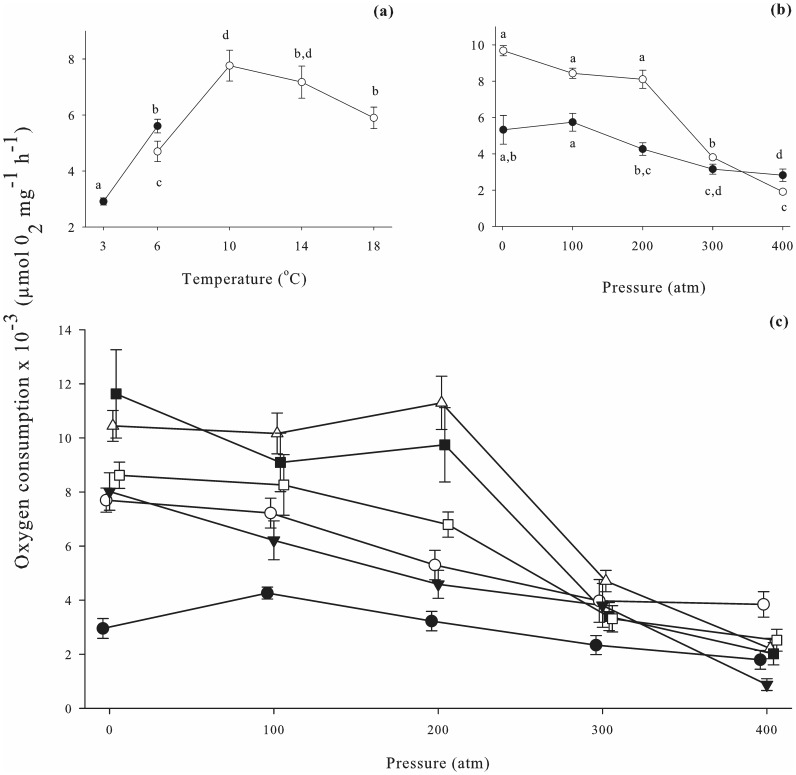
Oxygen consumption in hatching juvenile *Buccinum undatum* from the Solent (UK) and Breiðafjörður (Iceland). (a) Effects of temperature on oxygen consumption. Data for each temperature is averaged from 5 pressures (1, 100, 200, 300, 400 atm). Closed circles, Breiðafjörður data; open circles, Solent data. General Linear Model ANOVA indicated oxygen consumption to be significantly affected by temperature (*p≤*0.001); different letters indicate values that are significantly different. For each data point n = 45. (b) Effects of hydrostatic pressure on oxygen consumption. Data for each pressure is averaged across temperatures (6, 10, 14, 18°C for Solent samples; 3, 6°C for Breiðafjörður samples). Closed circles, Breiðafjörður data; open circles, Solent data. Oxygen consumption was significantly affected by pressure (*p≤*0.001); different letters indicate values that are significantly different. For each data point n = 54. (c) Change in oxygen consumption with pressure, at 6 temperatures. Closed circles, 3°C Breiðafjörður; open circles, 6°C Breiðafjörður; closed triangles, 6°C Solent; open triangles, 10°C Solent; closed squares, 14°C Solent; open squares, 18°C Solent. Oxygen consumption was significantly affected by temperature - pressure interactions (*p≤*0.001); see table 2 for *post hoc* analysis (Sidak simultaneous test). For each data point, n = 9. Data were analysed using General Linear Model ANOVA. Error bars display standard error.

Analysis indicated respiration rates in both veligers and hatching juveniles to be significantly affected by temperature (*p≤*0.001) (table 1, figs. 1a&2a). In veligers, a trend was observed at 1 atm of oxygen consumption increasing as temperature increased in samples from Breiðafjörður, but decreasing with increasing temperature in samples from the Solent. In juveniles, the trend was for oxygen consumption to decrease as temperature dropped, across both populations. When all pressures were averaged for each temperature, *post hoc* analysis (*p≤*0.05) indicated several groupings of similar temperatures for both veligers and juveniles (figs. 1a&2a).

**Table 1:** General Linear Model ANOVA results.

		Veliger		Hatching juvenile	
	n, df	F statistic	*p*-value	F statistic	*p*-value
**Temperature**	45, 5	30.14	≤0.001†††	30.63	≤0.001†††
**Pressure**	54, 4	0.94	0.440	105.01	≤0.001†††
Temperature* pressure	9, 20	0.56	0.934	4.42	≤0.001†††

Analysis testing the effects of temperature, pressure and temperature – pressure interactions on oxygen consumption in veliger and hatching juvenile *Buccinum undatum* from the Solent (UK) and Breiðafjörður (Iceland). Significance level is indicated by asterisks, † *p≤*0.05; †† *p≤*0.01; ††† *p≤*0.001. n = number of replicates, df = degrees of freedom.

### Effects of Pressure on Respiration Rates

Analysis indicated pressure to significantly affect respiration rates in all hatching juveniles (*p≤*0.001), but not in veligers (*p = *0.440) (table 1, figs. 1b&2b). A trend was observed of oxygen consumption to decrease with increasing pressure in juveniles from both Breiðafjörður and the Solent. When all temperatures were averaged for each pressure, *post hoc* analysis (*p≤*0.05) indicated hatching juveniles to be significantly affected by pressures of 200 atm or more (when compared to 1 atm). *Post hoc* analysis indicated there to be no difference in oxygen consumption in veligers between any pressures (*p≤*0.05).

### Temperature and Pressure Interaction Effects on Respiration Rates

Interaction effects of temperature and pressure were found to significantly impact oxygen consumption in hatching juveniles (*p≤*0.001), but not in veligers (*p = *0.934) (table 1, figs. 1c&2c). *Post hoc* analysis (*p≤*0.05) indicated there to be no difference in oxygen consumption in veligers between 1 atm, and any other pressure at any temperature (table 2a). In hatching juveniles, *post hoc* analysis (*p≤*0.05) indicated that the effects of increasing pressure on oxygen consumption were reduced with decreasing temperature (table 2b). In veligers, the largest fluctuation in respiration rates across all pressures was observed at 6°C (S), where oxygen consumption ranged from 0.012 (±0.004) µmol O_2_ mg^−1^ h^−1^ to 0.017 (±0.009) µmol O_2_ mg^−1^ h^−1^. The smallest difference was at 3°C (N) [range 0.004 (±0.002) to 0.005 (±0.002) µmol O_2_ mg^−1^ h^−1^]. In hatching juveniles, the largest change in oxygen consumption across all pressures occurred at 14°C (S) [range 0.002 (±0.001) to 0.012 (±0.005) µmol O_2_ mg^−1^ h^−1^] and the smallest was again at 3°C (N) [range 0.002 (±0.001) to 0.004 (±0.001) µmol O_2_ mg^−1^ h^−1^].

**Table 2:** *Post hoc* analysis (Sidak simultaneous test).

(a) *Veliger*		Pressure (atm)							
		100		200		300		400	
Temperature (°C)		T	*p-*value	T	*p-*value	T	*p-*value	T	*p-*value
**The Solent, UK**	**18**	0.167	1.000	0.588	1.000	−0.036	1.000	−0.571	1.000
	**14**	−0.768	1.000	−0.924	1.000	−1.344	1.000	−1.631	1.000
	**10**	−0.512	1.000	−0.517	1.000	−0.731	1.000	−0.177	1.000
	**6**	−1.341	1.000	−2.004	1.000	−0.652	1.000	0.246	1.000
**Breiðafjörður, Iceland**	**6**	−0.674	1.000	0.053	1.000	−1.117	1.000	−0.459	1.000
	**3**	−0.052	1.000	0.140	1.000	−0.765	1.000	−0.210	1.000
**(b) ** ***Juveniles***		**Pressure (atm)**							
		**100**		**200**		**300**		**400**	
**Temperature (°C)**		**T**	***p-*** **value**	**T**	***p-*** **value**	**T**	***p-*** **value**	**T**	***p-*** **value**
**The Solent, UK**	**18**	−0.590	1.000	−1.620	1.000	−5.570	<0.001†††	−6.860	<0.001†††
	**14**	−1.800	1.000	−1.310	1.000	−7.490	<0.001†††	−9.700	<0.001†††
	**10**	−0.250	1.000	0.540	1.000	−5.220	<0.001†††	−8.840	<0.001†††
	**6**	−1.746	1.000	−3.426	1.000	−4.706	0.002††	−9.530	<0.001†††
**Breiðafjörður, Iceland**	**6**	−0.459	1.000	−2.375	1.000	−4.080	0.026†	−4.075	0.027†
	**3**	1.786	1.000	0.379	1.000	−0.995	1.000	−1.970	1.000

The effects of pressure on oxygen consumption (when compared to 1 atm) across 6 temperatures (3 to 18°C) in (a) veliger and (N) hatching juvenile *Buccinum undatum* from the Solent (UK) and Breiðafjörður (Iceland).

Significance level is indicated by asterisks, † *p≤*0.05; †† *p≤*0.01; ††† *p≤*0.001.

n = 9.

T = test statistic.

## Discussion

### Cold Water Preference in Buccinidae?

Historically, neogastropods are believed to have a cold, deep-water origin, first appearing in fossil record on the ‘deep’ outer shelf during the mid cretaceous period [11], [35], [36]. Since then, they have evolved over millions of years to inhabit every corner of the oceans, across the full bathymetric and latitudinal range [37]. In particular within the Buccinidae, a distinct cold-water preference remains evident today. Species abundance is greatest in sub-polar and temperate areas (http://iobis.org/mapper/), and many species (including *B. undatum*) with ranges covering temperate or sub-tropical climates, show preference for cold water in their breeding cycle, with spawning and development occurring when annual water temperatures are at their lowest [39], [41], [49]. Recent work on thermal acclimation indicates that although a species can shift the limits of its thermal tolerance range to colonise new environments, optimal performance rarely acclimates to changes in environmental temperature [50,51]. It is therefore likely that the historical low-temperature bias observed during breeding in this family may remain beneficial for optimal performance. Amongst others, cold-water spawning and development in neogastropods have been observed in species from the Buccinidae, Conidae, Fasciolariidae and Muricidae [41], [49]. Our results indicate that in *B. undatum* this historical cold-water preference is particularly prevalent during early development. In juveniles, oxygen consumption scaled with temperature, as has been observed on many previous occasions for invertebrates [24], [32], [33], [52]–[55]. In veligers, in comparison, oxygen consumption increased with temperature until it exceeded 6°C, at which point it reduced, indicating thermal optima, and potentially also thermal maxima, to be lower at this developmental stage. These results are also supported by observations which indicate *B. undatum* to have a narrower thermal tolerance range during development than during adult life [40]. For each population, the highest oxygen consumption readings were taken at 6°C, indicating thermal optima may be relatively constant across the distribution range regardless of habitat temperature. The thermal optima we observed, also coincide with the minimum, and most successful, temperature at which complete intracapsular development occurs in *B. undatum* at the southern end of their distribution [40]. Northern populations, in comparison, and those which are historically adapted to sub-polar or polar climates, are also able to develop at temperatures below this [43], [56].

### Thermal and Hyperbaric Effects on Physiological Thresholds and the Importance of Complexity

Temperature and pressure have both frequently been argued to be major factors affecting physiology in invertebrates, thus playing a pivotal role in their distribution patterns [21], [23], [24], [50], [57]. Growth, survival and developmental success may all be affected by these factors, and internally, all membrane based processes have the potential to be disrupted by them [21], [23], [58], [59]. Organism complexity plays a key role in determining the intensity of such impacts, in particular in response to pressure. Quite simply, as an organism develops its complexity increases, making it more susceptible to the negative effects of pressure and often narrowing its tolerance window. Studies carried out on echinoderms indicate that when individuals are in their most complex form (i.e. adults) pressure tolerance is much lower than during development [13]. The effects of both temperature and pressure have also been shown to vary during development in invertebrates [16], [30], [60], [61].

While both temperature and pressure create substantial physiological obstacles for marine invertebrates, when comparing the effects of the two variables, temperature is commonly observed to be the dominant factor. Changes in temperature continuously affect invertebrates [e.g. 50,57,62], but past authors have demonstrated the effects of pressure on shallow-water invertebrates (crustaceans and echinoderms) to only become significant when equivalent to 2000 m or more [24], [31], [33]. The results of our study support these past investigations; temperature significantly affected veligers and juveniles, whereas pressure only affected juveniles, and only when greater than 200 atm. These findings also show similar ontogenetic shift in pressure tolerance as have been previously reported [16], [30], with juveniles being more susceptible than veligers.

### Polar Climates; the Optimum Environment for Deep-sea Invasion?

Several theories exist regarding the evolutionary expansion of ocean biodiversity. It is generally accepted that recurrent bathymetric colonisations and speciations have occurred, primarily via isothermal water columns. Ocean temperature profiles have, however, varied across geological periods, and with this, shifts in biodiversity. For example, well established patterns indicate latitudinal expansions in both shallow-water and deep-sea species diversity, with overall decreases from the tropics to the poles [5], [8], [9]. Such gradients are believed to have commenced in line with global cooling events such as that in the early Cenozoic, when ocean waters ceased to be homogenous, potentially limiting bathymetric migrations, and have been linked to the onset of seasonally fluctuating food supplies [6], [7]. Fossil records also show increases in speciations and biodiversity following glacial retreat. Theories suggest opportunistic taxa may go through rapid adaptive radiation in order to fill empty niches [63]. Such opportunistic taxa include species, which have been hypothesised to survive glacial advances through migration to deeper waters [64]. Conflicting views exist regarding the expansion of extant fauna of deep-sea chemosynthetic environments. While previous studies have suggested deep-sea vents and seeps to have been colonised by fauna from shallow-water seeps, pre-adapted to such environments [65], recent works instead suggest invasions primarily occur latitudinally from adjacent deep-sea environments, indicating isothermal water columns to contribute minimally in facilitating such invasions [66], [67].

Here, we focus on the widely accepted hypothesis of bathymetric migrations occurring via isothermal water columns [10]–[16]. While during past geological periods (e.g. the late Mesozoic and early Cenozoic), ocean waters were warm and relatively homogenous throughout, today ocean temperatures average ∼4°C below 1000 m [10]–[13], [68]. Regions where surface waters are of a similar temperature to this, make up the largest areas of isothermal waters. If we consider bathymetric colonisations and speciations to primarily occur via such waters, these areas are therefore the most likely locations for modern bathymetric migrations.

Since the effects of temperature and pressure go hand in hand, these two variables should be evaluated collectively when considering the potential for deep-water colonisation. Our results imply *B. undatum* to theoretically be capable of surviving the combined thermal and hyperbaric conditions characteristic of the deep sea. While ontogenetic shifts were evident in both pressure and temperature thresholds, contrary to past studies [e.g. 15,24,30,32,33], our results indicate pressure sensitivity to decrease at low temperature, with individuals being capable throughout development of withstanding pressures equivalent to at least 4000 m at the lowest experimental temperature of 3°C.

Previous studies indicate theoretical depth penetration by shallow-water crustaceans and echinoderms to be possible at temperatures close to the upper limits of, or above, those experienced in their natural environment [e.g. 12,15,24,30,32,33]. The antagonistic effects of temperature and pressure imply that increases in one, may compensate to some degree for increases in the other. For example, membrane fluidity is affected by both variables but while high pressure decreases fluidity, high temperature increases it. If both variables increase simultaneously therefore, membrane fluidity may be minimally affected [for reviews see 21,23]. Environmental changes, however, rarely follow this pattern, and adaptations often occur as a result of changes in just one variable. Since similar adaptations develop as a result of increased pressure or decreased temperature, organisms, which have already adapted to low temperatures, for example, may be pre-adapted to some degree for high pressure. Growth at low temperatures has been shown to increase pressure resistance in the bacterium *Escherichia coli* [69], [70]. Similarly, studies examining the effects of pressure on shallow-water echinoderm embryos, indicate pressure tolerance at native temperatures to be greater in polar species [30] than in temperate species [31]. We suggest that in other marine invertebrates a similar phenomenon occurs, with cold-adapted populations being less affected by increases in pressure than warm-adapted populations. Under this scenario, as ocean surface waters continue to warm, *B. undatum* and similar cold-adapted species may migrate along isotherms, taking refuge in deeper waters which appear optimal for their physiology and using this as a mechanism to survive. Such migrations have already been reported in a range of cold-water marine fish, in response to warming surface waters [26]–[28]. Similar migrations have also been suggested in response to decreasing temperatures; evidence of glacial refugia indicates some species to have previously seeked refuge in deeper waters in order to avoid advancing ice sheets [14], [64]. The relationship between depth range and latitude often observed in marine invertebrates gives support for this, with several species showing patterns of increasing depth limits at high latitudes [71]. While *B. undatum* is known to exist in waters shallower than 250 m [47], to our knowledge, records detailing variations in depth distribution across the species range are incomplete and further study is needed to determine whether this species follows the same pattern.

While theoretically, this study shows *B. undatum* to be capable of cold, deep-water penetration, the shallow-water distribution of this species suggests factors other than temperature and pressure may currently limit its distribution. Species range limits are set by a combination of many biological, physical and chemical factors [15], [29]. Such factors include food availability, predation, competition, habitat and water chemistry [15], [29]. Alternatively, the thermal and hyperbaric tolerance observed throughout early ontogeny in this species may not be representative of that which it can tolerate throughout the duration of its entire life history; an important aspect that remains subject to future study, including the challenge of long-term maintenance of invertebrates under high hydrostatic pressures.

Our study provides evidence that if thermally acclimated throughout development, high-pressure tolerance is possible at the low temperatures typical of deep-sea environments. Although questions remain regarding the potential for complete egg mass development and successful growth to adult life under high pressure, or the impact of additional factors such as species interactions, our findings suggest populations of *B. undatum* from the northern end of the distribution to be theoretically capable of submergence into deep water through cold isothermal water columns typical of those found in Polar Regions. Cold-acclimated juveniles from the southern end of the distribution also showed greater tolerance to pressure (to 400 atm), relative to warm-acclimated individuals from the same population (to 200 atm).

Thermal acclimation is an important mechanism affecting physiological scope, and past studies have indicated shifts in thermal range as a result of acclimation, which in turn affects performance at a given temperature [51,72]. The present study increases our knowledge of the physiological effects of temperature and pressure on invertebrates and highlights the importance of thermal acclimation in experimentation, giving fresh insight into how evolutionary colonisations via polar isothermal water columns may have been achieved.
